# Carbidopa/levodopa induced severe vitamin B6 deficiency leading to symptomatic transfusion dependent microcytic Anemia

**DOI:** 10.1093/omcr/omaf191

**Published:** 2026-04-28

**Authors:** Byung K Lee, Jaime P Gastwirt

**Affiliations:** Department of Medicine, Naval Medical Center San Diego, 34800 Bob Wilson Drive, San Diego, CA 92134, United States of America; Department of Hematology & Oncology, Naval Medical Center San Diego, 34800 Bob Wilson Drive, San Diego, CA 92134, United States of America

**Keywords:** microcytic Anemia, vitamin B6 deficiency, carbidopa/levodopa, Parkinson’s disease

## Abstract

Parkinson’s Disease is on the rise over the coming decades with expectations that more patients will be prescribed levodopa therapy. This case presents a patient with Parkinson’s Disease who presented with severe symptomatic microcytic anemia. He was transfusion dependent during his extensive work up that occurred during both inpatient hospitalization and outpatient hematology visits. This was negative for iron deficiency, infectious etiology, hemolysis, active bleeding, bone marrow failure, myelodysplasia or hematologic malignancy. However, patient was found to have a critically low vitamin B6 level. Following initiation of vitamin B6 supplementation, patient’s anemia resolved and was no longer transfusion dependent.

## Background

With an aging population and increased disease awareness, the incidence of Parkinson’s Disease is expected to rise over the coming decades [1]. Based on the American Academy of Neurology Dopaminergic Therapy for Motor Symptoms in Early Parkinson’s Disease Practice Guideline, levodopa therapy is one of the first-line recommended treatments for early Parkinson’s Disease [2]. As such, it is expected that more patients will be prescribed levodopa for pharmacological management. This case presents a patient with transfusion-dependent microcytic anemia secondary to severe vitamin B6 deficiency from oral carbidopa/levodopa.

## Case report

Patient is a gentleman in his early 60’s with a history of Parkinson’s Disease, chronic kidney disease grade 3a/A2 and parathyroid adenoma presented to the hematology clinic for a persistent transfusion dependent microcytic anemia. Parkinson’s Disease was diagnosed one year prior to his initial presentation. Patient had classical features of unilateral resting tremor, masked facies, and prominent bradykinesia and rigidity on neurology examination. Magnetic resonance imaging of the brain prior to the visit did not identify any confounding factors. There was no concern for atypical Parkinsonism syndrome or Amyotrophic Lateral Scelerosis-Parkinsonism Dementia phenotype. Patient was started on carbidopa/levodopa 25 mg—100 mg three times daily with excellent response just at one month of therapy.

Roughly eight months into therapy, the patient presented to the emergency department with severe symptomatic anemia to include sharp substernal exertional chest pain, shortness of breath, and lightheadedness. Index vital signs showed a temperature 36.4°C, heart rate 122 beats/minute, respiration 18 breaths/minute, and blood pressure 141/91 mmHg. Initial evaluation in the emergency room revealed severe microcytic anemia with a hemoglobin of 3.8 g/dl (ref. 13.8–17.0 g/dl) and mean corpuscular volume (MCV) of 72.5 fL (ref. 82.0–99.0 fL). This was a significant drop from a hemoglobin of 13.0 g/dl prior to initiation of carbidopa/levodopa. Subjectively, the patient reported a deteriorating functional status over the past six months. Although the patient endorsed dark stools within the past month, there was no evidence of frank bleeding on rectal examination, and he was on oral iron supplementation. He was admitted for expedited inpatient work up. During his hospitalization, the patient required a total of five units of packed red blood cells and received epoetin alfa before discharge.

A comprehensive inpatient and outpatient diagnostic work-up ensued. Extensive laboratory work-up was ultimately negative—no evidence of iron deficiency, vitamin B12, vitamin D or folate deficiency, heavy metal toxicity, hemoglobinopathy, zinc/copper abnormalities, thyroid dysfunction, monoclonal gammopathy, parvovirus, HIV, hepatitis, or hemolysis. While patient had evidence of mild proteinuria with a urine protein-to-creatinine ratio of 1.78 g/day, this had been chronic and there was no significant progression of his kidney disease based on serum creatinine and estimated glomerular filtration rate (eGFR). Nephrology evaluation assessed the most likely etiology as hypertension and did not feel a renal biopsy would urgently change management. He completed an upper and lower endoscopic evaluation with Gastroenterology that was negative for an overt source of gastrointestinal bleed. Bone marrow biopsy revealed normocellular marrow with maturing trilineage hematopoiesis, increased iron storage and no significant fibrosis. Bone marrow morphology, flow cytometry and cytogenetics were essentially normal—no evidence of bone marrow failure, myelodysplastic syndrome or hematologic malignancy. Most notably, however, was a severely decreased serum vitamin B6 level at 1.2 mcg/L (reference range: 3.4–65.2 mcg/L). While vitamin B6 deficiency can be associated with a sideroblastic anemia, upon re-review of bone marrow biopsy, no ringed sideroblasts were noted per pathology. The complete diagnostic work-up results are portrayed in Table 1.

**Table 1:** Comprehensive work up and respective results gathered during the diagnostic phase of evaluating the patient’s microcytic anemia. Most notable for the low vitamin B6 levels.

LAB	RESULTS	REFERENCE
Hematology Studies		
White Blood Cells	4.7 × 10^3^/ul	4.0–10.5 × 10^3^/ul
Hemoglobin	6.3 g/dl	13.8–17.0 g/dl
Hematocrit	20.80%	40.0–50.0%
Mean Corpuscular Volume	77.1 fl	82.0–99.0 fl
Mean Corpuscular Hemoglobin	25.3 pg	28.0–33.0 pg
Mean Corpuscular Hemoglobin Concentration	30.3 g/dl	32.0–36.0 g/dl
Red Cell Distribution Width	23.60%	11.5–14.0%
Platelet Count	231 × 10^3^/ul	150–450 × 10^3^/ul
Reticulocyte	0.56%	0.50–2.20%
Reticulocyte Index	0.14	
Hemoglobin Fractionation	Hemoglobin pattern and concentration consistent with a delta chain variant	
Peripheral Blood Smear	Hypochromic, microcytic anemia without sideroblasts	
Chemistry Studies		
Sodium	141 mmol/l	136–145 mmol/l
Potassium	4.0 mmol/l	3.5–5.1 mmol/l
Chloride	106 mmol/l	98–107 mmol/l
Bicarbonate	24 mmol/l	22–32 mmol/l
Blood Urea Nitrogen	23 mg/dl	6.0–20.0 mg/dl
Serum Creatinine	1.3 mg/dl	0.7–1.2 mg/dl
Calcium	10.5 mg/dl	8.9–10.4 mg/dl
Phosphorus	3.3 mg/dl	2.5–4.5 mg/dl
Magnesium	2.0 mg/dl	1.7–2.6 mg/dl
Protein, Total	6.8 g/dl	6.6–8.7 g/dl
Albumin	3.8 g/dl	3.5–5.0 g/dl
Total Bilirubin	0.8 mg/dl	0.15–1.00 mg/dl
Alkaline Phosphatase	93 U/l	40–129 U/l
Alanine Aminotransferase	5 U/l	17–63 U/l
Aspartate Aminotransferase	8 U/l	12–39 U/l
Vitamin B12	672 pg/ml	232–1245 pg/ml
Folate	6.1 ng/ml	> 5.0 ng/ml
Methymalonate Acid	616 nmol/l	0–378 nmol/l
Vitamin B6	1.2 mcg/l	3.4–65.2 mcg/l
25-Hydroxyvitamin D	34.0 ng/ml	30–100 ng/ml
Haptoglobin	116 mg/dl	30–200 mg/dl
Lactate Dehydrogenase	128 U/l	135–225 U/l
Parathyroid Hormone	143 pg/ml	15–65 pg/ml
Parathyroid Hormone-related Protein	< 2.0 pmol/l	< 2.0 pmol/l
Erythropoietin	89.7 mlU/ml	2.6–18.5 mIU/ml
Thyroid Stimulating Hormone	0.24 ulU/ml	0.27–4.20 uIU/ml
Free T4	0.99 ng/dl	0.89–1.76 ng/dl
Serum Protein Electrophoresis		
Kappa Free Light Chain	49.3 mg/l	3.3–19.4 mg/L
Lambda Free Light Chain	34.7 mg/l	5.7–26.3 mg/L
Kappa/Lambda Free Light Chain Ratio	1.42	0.26–1.65
Protein Monoclonal Spike	Negative	
Iron Studies		
Iron	214 ug/dl	59–158 ug/dl
Total Iron Binding Capacity	217 ug/dl	280–504 ug/dl
Iron Saturations	99%	15–69%
Ferritin	1485.78 ng/ml	30–400 ng/ml
Heavy Metals		
Mercury	< 1.0 mcg/l	0.0–14.9 mcg/l
Lead	<1.0 ug/dl	<1.0 ug/dl
Copper	79 ug/dl	69–132 ug/dl
Copper, Red Blood Cells	0.49 ug/ml	0.50–1.00 ug/ml
Arsenic	2 mcg/l	0–9 mcg/l
Cadmium	< 0.5 mcg/l	0.0–1.2 mcg/l
Zinc	58 ug/dl	44–115 ug/dl
Microbiology Studies		
Parvovirus B19, DNA PCR	Negative	
Human Immunodeficiency Virus 1/2 Antibody Rapid Screen	Negative	
Bone Marrow Biopsy		
Bone Marrow Morphology Analysis	Normocellular marrow with maturing trilineage hematopoiesis and relative erythroid hyperplasia with left shift. Markedly increased storage iron. No significant fibrosis.	
Bone Marrow Flow Cytometry	No significant immune-genotypes abnormalities detected. Blasts are not increased. Polyclonal B Cells. Immunophenotypically unremarkable T cells.	
Whole Genome Chromosome Single Nucleotide Polymorphism (SNP) Microarray	Normal	
Cytogenetic Analysis	Four of twenty metaphase cells (20%) analyzed with loss of the Y chromosome. No other chromosome abnormalities were observed. Loss of Y increases with age but much greater in males with MDS, MPD, AML, or lymphoproliferative disorders	
Next Generation Gene Sequencing	No clinically significant variants were detected	
Endoscopy		
Esophagogastroduodenoscopy + Colonoscopy	No source of anemia identified	

Peripheral blood demonstrated a chronic hypo-proliferation microcytic anemia without clinical or laboratory evidence of iron deficiency, infectious etiology, hemolysis, active bleeding, bone marrow failure, myelodysplasia or hematologic malignancy. Given the remarkable finding of a severely low vitamin B6 level at 1.2 mcg/L, the patient’s microcytic anemia was ultimately attributed to a critical vitamin B6 deficiency.

**Figure 1:**
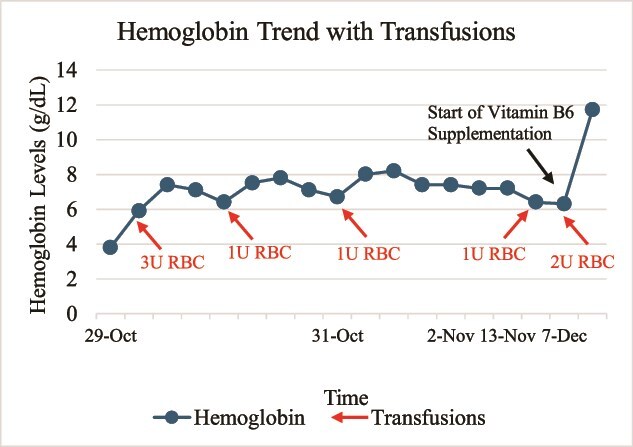
Trend of hemoglobin over the span of two months requiring a total of 8 units of packed red blood cells. Once vitamin B6 supplements were started in the beginning of December, there was a significant improvement in hemoglobin and no longer requiring transfusions.

For two months during his extensive work up, patient remained transfusion dependent—requiring a total of 8 units of packed red blood cells over that time frame. Once the patient was found to be vitamin B6 deficient, he was initiated on pyridoxine 50 mg once daily after consultation with the patient’s primary neurologist. After one year of starting vitamin B6 supplementation, the patient was no longer anemic with hemoglobin levels returning to normal > 13.0 g/dl and vitamin B6 levels within normal limits. He has not required additional transfusions to date and remains asymptomatic. Figure 1 depicts the timeline of the patient’s clinical course with the dramatic improvement in hemoglobin after supplement initiation.

## Discussion

Literature review reveals an association between vitamin B6 deficiency and carbidopa/levodopa. Modica et al. performed a systematic meta-analysis reviewing abnormal vitamin B6 levels in patients with Parkinson’s Disease. The study found that out of 65 subjects on intestinal gel carbidopa/levodopa and 73 subjects on oral carbidopa/levodopa, 44.6% and 30.1% of subjects, respectively, were vitamin B6 deficient [3]. Vitamin B6, also known as pyridoxine, plays a vital role in heme biosynthesis. Pyridoxal 5′ phosphate, the activated form of pyridoxine, is an essential cofactor for aminolevulinic acid synthase which drives the initial steps of heme synthesis to make 5-aminolevulinic acid [4]. Additionally, pyridoxal 5′ phosphate is critical in the metabolism of levodopa to dopamine via decarboxylation [5].

The consequences of vitamin B6 deficiency from carbidopa/levodopa have been reported through multiple case reports. Two patients diagnosed with Parkinson’s Disease who were initiated on the intestinal gel formulation developed polyneuropathy as early as 4 weeks proven on magnetic resonance neurography and found to have low vitamin B6 levels [6]. Another case report discussed a patient with a six-year history of Parkinson’s Disease on oral carbidopa/levodopa, who presented with refractory seizures secondary to vitamin B6 deficiency [5]. Finally, a case report from Japan documented a patient with Parkinson’s Disease on carbidopa/levodopa intestinal gel who developed severe microcytic anemia associated with undetectable vitamin B6 levels requiring multiple transfusions [7].

This is the first case in the United States of transfusion-dependent microcytic anemia secondary to severe vitamin B6 deficiency from oral carbidopa/levodopa. Much like isoniazid therapy, clinicians should preemptively survey for vitamin B6 deficiency and pyridoxine supplementation should be considered when prescribing carbidopa/levodopa.

## DoD Disclaimer

The opinions and assertions contained herein are those of the authors and do not reflect those of the United States Navy or the Department of Defense.
